# Occurrence and Temporal Variation of Technology-Critical Elements in North Sea Sediments—A Determination of Preliminary Reference Values

**DOI:** 10.1007/s00244-022-00929-4

**Published:** 2022-04-26

**Authors:** Ole Klein, Tristan Zimmermann, Anna Ebeling, Madita Kruse, Torben Kirchgeorg, Daniel Pröfrock

**Affiliations:** 1 Institute of Coastal Environmental Chemistry, Inorganic Environmental Chemistry, Helmholtz-Zentrum Hereon, Max-Planck Str. 1, 21502 Geesthacht, Germany; 2 Department of Chemistry, Inorganic and Applied Chemistry, Universität Hamburg, Martin-Luther-King-Platz 6, 20146 Hamburg, Germany; 3 Department Mechanical Engineering, HTWG Hochschule Konstanz, Alfred-Wachtel-Straße 8, 78462 Konstanz, Germany; 4 Marine Sciences Department, Marine Chemistry Laboratory – Shipping and Environment, Marine Sediments Section, Bundesamt für Seeschifffahrt und Hydrographie (BSH), Wüstland 2, 22589 Hamburg, Germany

## Abstract

As interest in the investigation of possible sources and environmental sinks of technology-critical elements (TCEs) continues to grow, the demand for reliable background level information of these elements in environmental matrices increases. In this study, a time series of ten years of sediment samples from two different regions of the German North Sea were analyzed for their mass fractions of Ga, Ge, Nb, In, REEs, and Ta (grain size fraction < 20 µm). Possible regional differences were investigated in order to determine preliminary reference values for these regions. Throughout the investigated time period, only minor variations in the mass fractions were observed and both regions did not show significant differences. Calculated local enrichment factors ranging from 0.6 to 2.3 for all TCEs indicate no or little pollution in the investigated areas. Consequently, reference values were calculated using two different approaches (Median + 2 median absolute deviation (M2MAD) and Tukey inner fence (TIF)). Both approaches resulted in consistent threshold values for the respective regions ranging from 158 µg kg^−1^ for In to 114 mg kg^−1^ for Ce. As none of the threshold values exceed the observed natural variation of TCEs in marine and freshwater sediments, they may be considered baseline values of the German Bight for future studies.

**Graphical Abstract:**

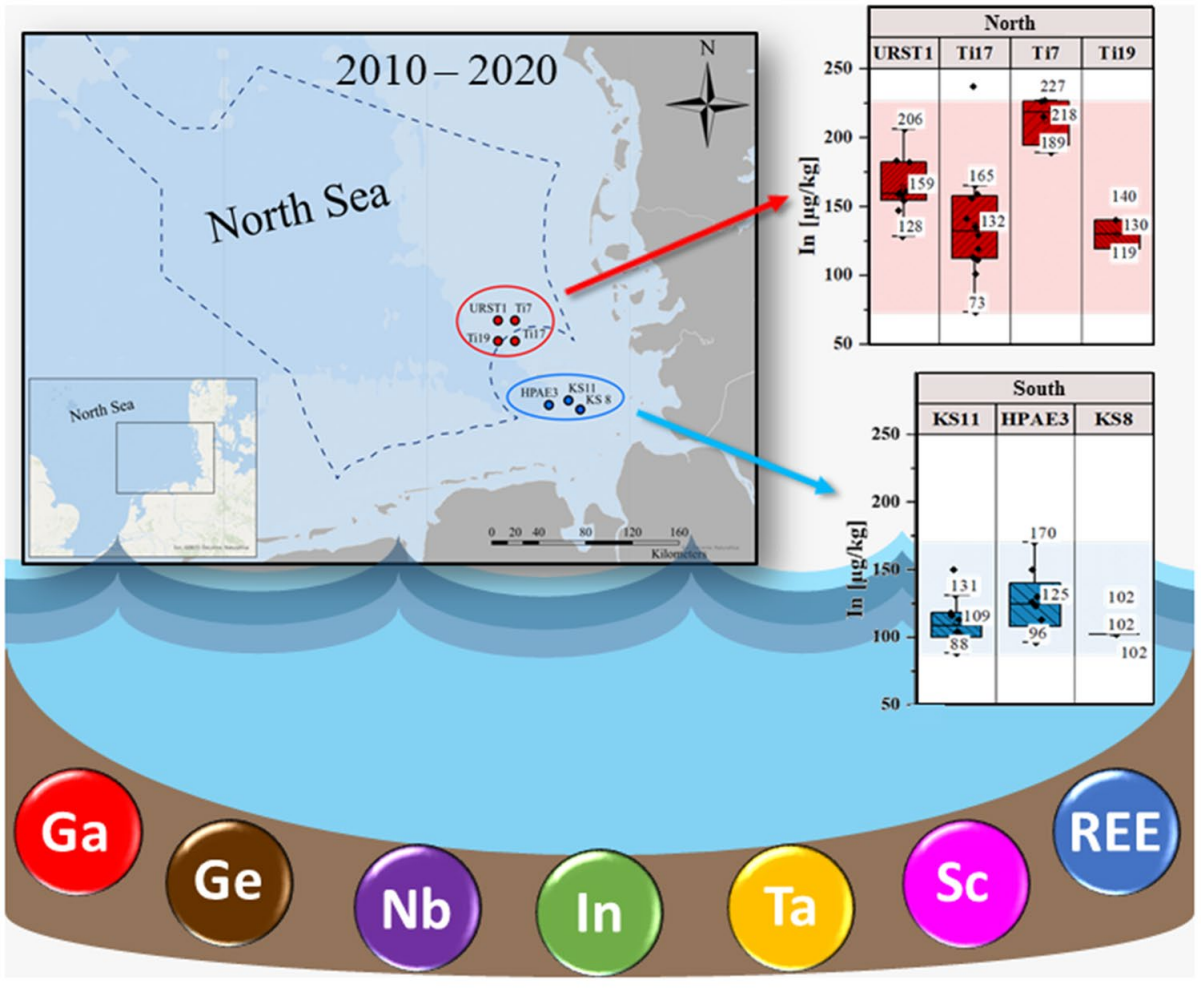

**Supplementary Information:**

The online version contains supplementary material available at 10.1007/s00244-022-00929-4.

The assessment of metals and metalloids in the environment is of great concern for many legislators to ensure a good environmental status. Especially for marine environments, legal directives regulate the maximum values of pollutants in aquatic systems (e.g., Water Framework Directive 2000/60/EC, Marine Strategy Framework Directive 2008/56/EG). Moreover, transboundary committees urge actions for the protection of the good environmental status, e.g., stated in the sustainable development goals (SDGs) of the United Nations or the Oslo-Paris-Convention (OSPAR). However, besides the continuously monitored legacy pollutants (e.g., Ni, Cu, Zn, Cd, Hg, Pb), new potential contaminants like the so-called technology-critical elements (TCEs) (including Ga, Ge, Nb, In, REEs and Ta) may play an important role in the near future (Filella and Rodriguez-Murillo 2017; Filella and Rodushkin 2018; Klein et al. 2021; Nuss and Blengini 2018; Reese et al. 2020).

Even though heavy metals like Cu and Zn show extensively higher annual production amounts (e.g., in 2017 Cu: 2.0 10^7^ t/a vs. Nb: 6.7 10^4^ t/a), TCEs are considered emerging contaminants as they have similar pathways into the environment (Kelly et al. 2005). Especially given that many TCEs still have highly disrupted circular economies, in part due to low recycling rates, their impact on the environment may be of greater concern than other heavy metals (Barth et al. 2000; Bu-Olayan and Thomas 2020; Grandell et al. 2016; Hagelüken 2014; Kaya 2019; Ray et al. 2020; Romero-Freire et al. 2019). However, the assessment of TCEs is still challenging as on one hand only little is known about possible (eco)toxicological effects of these elements, and on the other hand, little information on specific environmental background values for most of the TCEs are available.

The ecotoxicological evaluation of environmental samples such as sediments is challenging. In addition to the mass fractions of possible pollutants, knowledge of other parameters such as pH, grain size, or biogeochemical processes in the respective regions is required. Hence, geochemical thresholds are often used as a basis for (legally binding) limit values of different pollutants and to allocate elevated concentrations (Reimann et al. 2018). Therefore, there is a high demand for data on background thresholds or background reference values of TCEs in environmental research (Lučić et al. 2021). In order to determine geological background threshold values, often the Median + 2 median absolute deviation (M2MAD) or the Tukey inner fence (TIF) method are used. The M2MAD method usually results in lower, thus more conservative, threshold values compared to the TIF method, whereas the TIF method provides very robust threshold values against possible outliers within the used datasets (Reimann et al. 2018).

Particularly in marine environmental analysis, sediment analysis is used to investigate possible trends in complex systems such as rivers, estuaries, or coastal regions (Ackermann et al. 1983; Deng et al. 2021; Leschber et al. 1985; Logemann et al. 2022; Reese et al. 2019). For this reason, sediment analysis is often used for the long-term assessment of contaminants of aquatic systems like the North Sea. However, in order to obtain comparable and standardized mass fractions of the individual elements, different normalization approaches are commonly applied. Isolation of the fine fraction by sieving (commonly either < 20 μm or < 63 μm) is regarded as a physical normalization reducing differences in the granulometric composition. Coarser particles, which usually do not bind anthropogenic contaminants and would therefore dilute their mass fractions, are removed from the sample. Subsequently, natural geochemical differences in sediment composition may be further corrected by the use of cofactors like the Al content (Ackermann et al. 1983; Federal Maritime and Hydrographic Agency (BSH) 2009; Federal Maritime and Hydrographic Agency (BSH) 2016; OSPAR/CEMP 2002). The North Sea is considered an area with high anthropogenic pressures such as shipping, offshore oil and gas production, offshore wind energy production, as well as inputs from its fluvial tributaries (e.g., Rhine, Elbe, Glomma, Weser, Ems) (Deng et al. 2021; Federal Ministry for the Environment Nature Conservation Nuclear Safety and Consumer Protection (BMUV) 2018; German Environment Agency (UBA) 2018; Reese et al. 2020; Reese et al. 2019). Today the environmental status of the North Sea is constantly monitored by national agencies with the aim of achieving a good environmental status in accordance with the EU Maritime Strategy Framework Directive. However, up to now some metals like Pb, Hg, and Zn are still above the defined threshold values, indicating possible anthropogenic pollution (Federal Maritime and Hydrographic Agency (BSH) 2016; Federal Ministry for the Environment Nature Conservation Nuclear Safety and Consumer Protection (BMUV) 2018). Even though elevated inputs of TCEs are considered possible, data on the TCE load of North Sea sediments are very scarce. Potential TCE input sources in this context are offshore industries and fluvial tributaries such as the Elbe, Weser, Ems, or Rhine (Deng et al. 2021; Kirchgeorg et al. 2018; Reese et al. 2020, 2019).

In this work, a time series of sediment samples of two different locations within the German North Sea were analyzed for their TCE mass fractions within the grain size fraction < 20 µm to determine the occurrence and temporal variation of TCEs in the North Sea. Furthermore, this study aims to define preliminary reference values for TCEs in these particular areas using the M2MAD and TIF approaches.

## Material and Methods

### Reagents and Standards

Preparatory laboratory work was conducted in class 10,000 or class 1,000 clean rooms. Only type I reagent grade water (> 18.2 MΩ cm), obtained from an ultrapure water system consisting of an Elix 3 module (Merck Millipore, Darmstadt, Germany), a Milli-Q element module (Merck Millipore, Darmstadt, Germany), and a Q-POD element (Merck Millipore, Darmstadt, Germany), was used. Used acids were either further purified by double sub-boiling in perfluoralkoxy polymer (PFA)-sub-boiling stills (DST-4000 & DST-1000, Savillex, Minnesota, USA) from analytical grade acids (HNO_3_ (65% *w*/*w*, Fisher Scientific GmbH, Schwerte, Germany), HCl (30% *w*/*w*, Carl Roth GmbH + Co. KG, Karlsruhe, Germany)) or were obtained in ultrapure quality (HBF_4_ (38% *w*/*w*, Chem-Lab, Zedelgem, Belgium)).

Single element standards (Carl Roth GmbH, Karlsruhe, Germany or Sigma-Aldrich, Missouri, USA) and custom-made multielement standards (all traceable to NIST standards) of different compositions (Inorganic Ventures, Christiansburg, USA) were used for the preparation of diluted standard solutions.

The reference marine sediment GBW 07313 (National Research Centre for Certified Reference Materials, Beijing, China), the reference stream sediment GBW 07311 (National Research Centre for Certified Reference Materials), and basalt reference material BCR-2 (United States Geological Survey Certificate of Analysis, Denver, USA) were used for method development and validation.

### Sampling

**Fig. 1:**
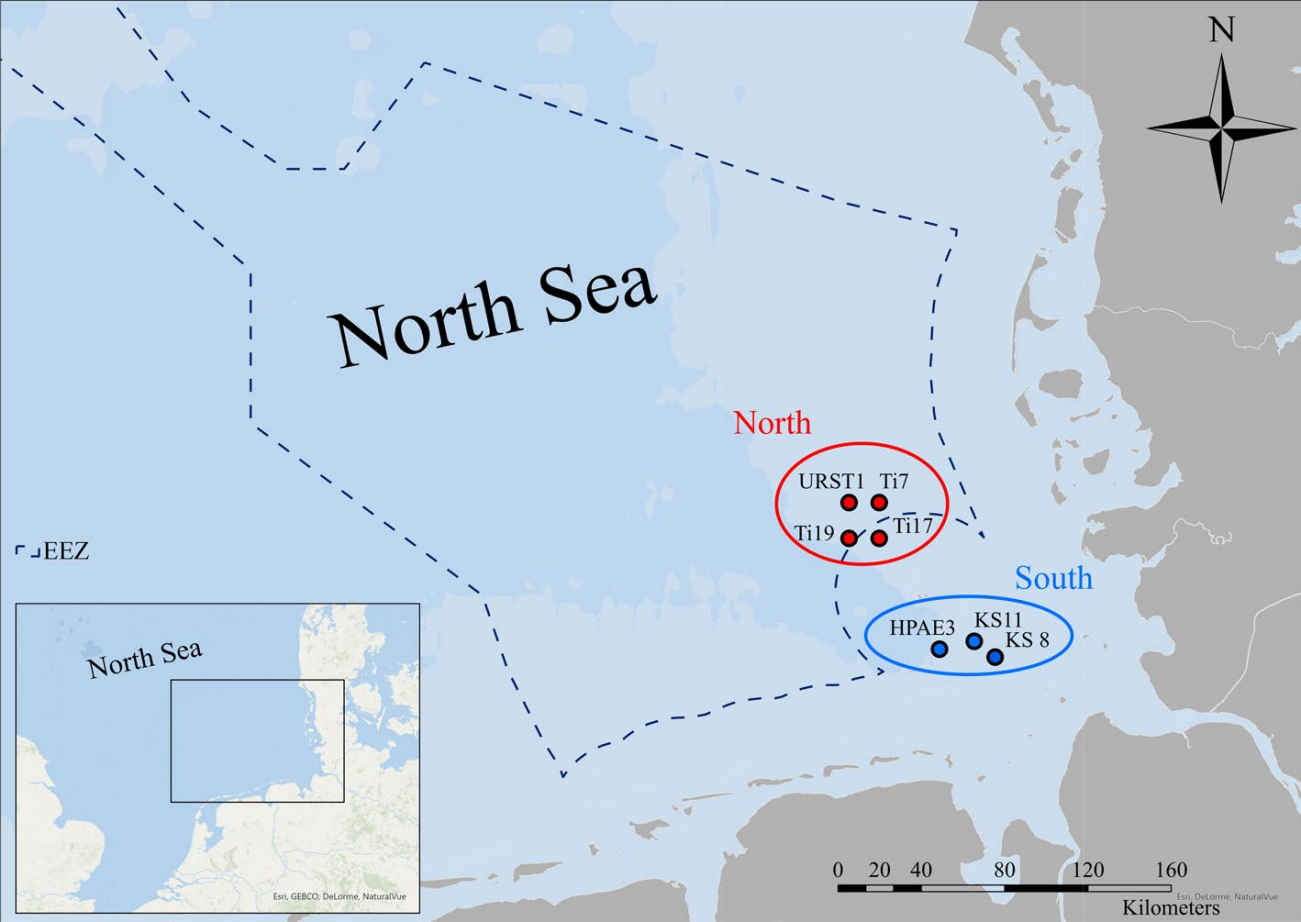
Overview of the sampling stations in the German Bight. Red dots correspond to stations of the North region while blue dots show the South region. The dashed line highlights the German *exclusive economic zone* (EEZ)

In the course of several sampling campaigns between 2010 and 2020, a total of 50 sediment samples were obtained. For each campaign, the sampling was performed by either the Bundesamt für Seeschifffahrt und Hydrographie (Federal Maritime and Hydrographic Agency (BSH)), the Helmholtz-Zentrum Hereon (Hereon) or in cooperation of both. Table 1 lists all sampling campaigns along with the sampling time, the respective research vessel, and the campaign code. All surface sediment samples were collected with a box corer. The top layer (approx. 2–3 cm) of one or more individual samples (depending on sediment type) were collected, pooled, and deep-frozen. For this study, a subset of seven sediment samples from each campaign was used. Samples of two different regions were analyzed: one close to the coast of Heligoland (South) and a second further offshore in the North Sea (North). A list of all samples including coordinates is given in the supplemental information Table A1 while Fig. 1 shows a map with the sample locations.

**Table 1:** Overview of all campaigns from which samples were included in this study

Campaign code	Research vessel	Administration	Sampling time
CE10001	Celtic Explorer	BSH	01–2010
CE11002	Celtic Explorer	BSH	01–2011
CE12002	Celtic Explorer	BSH	01–2012
PE364	Pelagia	BSH	01–2013
PE385	Pelagia	BSH	01–2014
CE15001	Celtic Explorer	BSH	02–2015
CE16011b	Celtic Explorer	BSH	09–2016
LP20160725	Ludwig Prandtl	Hereon	07–2016
CE17001	Celtic Explorer	BSH	01–2017
AT248	Atair	BSH	05–2017
AT261	Atair	BSH + Hereon	04–2018
AT275	Atair	BSH + Hereon	03–2019
LP20200629	Ludwig Prandtl	Hereon	06–2020

### Sample Digestion

Prior to any further sample preparation, each sample was analyzed for its grain size distribution using either a Laser Particle Sizer (Analysette 22, FRITSCH, Ida-Oberstein, Germany) or by a sieve analysis.

All sediment samples were freeze-dried (Christ Gefriertrocknungsanlagen, Osterode, Germany) and wet-sieved, using a cascade of polyamide sieves (RETSCH GmbH, Haan, Germany) and either a continuous-flow (Hereon) or a standard centrifuge (BSH) (Thermo Scientific™ Heraeus™ Biofuge™ Stratos™ Zentrifuge, Fisher Scientific GmbH, Schwerte, Germany) to obtain the < 20 μm grain size fraction. The wet-sieving was performed in a closed setup using 1 L of Milli-Q water. Leaching of analytes into the water used for sieving is negligible (5 × 10^−5^%–5 × 10^−9^% for all analytes considered) (Nham 2017). After wet-sieving triplicates of each sediment sample were digested via microwave-assisted acid digestion using either a MARS Xpress or a MARS 6 (CEM Corp., Kamp Lintfort, Germany) following the digestion protocol of Zimmermann et al. (2020): 50 mg of sieved sediment was digested using a mixture of 5 mL HNO_3_, 2 mL HCl, and 1 mL HBF_4_ at 180 °C for 300 min.

Two sediment reference materials GBW 07313 and GBW 07311 as well as the basalt reference material BCR-2 were digested in a similar manner. All samples were prepared in triplicates. Certified and measured values for the certified reference materials (CRMs) and respective recoveries for the quantified elements together with the respective measurement mode, *LOD,* and *LOQ* values of the method are given in supplemental information Tables A.2 and A.3.

**Table 2:** Comparison of the observed TCE mass fraction ranges (< 20 µm grain size fraction) of this study with other environmental sample matrices

	This study		Ohta et al. (2010)	Dolor et al. (2009)	Lučić et al. (2021)	Bačić et al. (2021)	Salminen et al. (2006)^1^	Rudnick and Gao (2003)
	North	South	coastal	coastal	riparian soil	lake	river	UCC
Sc/mg kg^−1^	8.1–18.1	8.8–18.2	1.56–67		2.5–18.9	0.6–15.4		13.1–14.9
Y/mg kg^−1^	11.9–31.5	14.2–31.6	2.5–34.1		4.8–46.2	0.42–41.7	25.65	19–23
Ga/mg kg^−1^	14–23	14–20	2.2–20.9	9–20	3–24.6	0.19–19.8		16.8–18.2
Ge/mg kg^−1^	1.4–2.3	1.2–1.9		1.6–2.2	0.22–2.3	0.01–1.5		1.3–1.5
Nb/mg kg^−1^	11.0–15.4	8.0–18.2	0.7–28.4	9–13	2.2–20.1	0.19–17.4		11–13
In/µg kg^−1^	73–237	88–170		130–240				48–64
La/mg kg^−1^	18.7–41.9	20.3–58.9	2.89–30.9		6.3–51.3	0.44–42.3	32.50	28–34
Ce/mg kg^−1^	45–88	46–120	7.01–59.2		12.3–106		66.60	56–67
Pr/mg kg^−1^	6.3–11.0	6.5–14.9	0.98–7.8		1.2–13.5		7.35	7.1
Nd/mg kg^−1^	24–40	25–54	4.01–28.5		5–53.6		28.25	25–29
Sm/mg kg^−1^	5.2–8.6	5.3–10.2	0.84–5.95		1.2–12		5.4	4.0–5.0
Eu/mg kg^−1^	1.08–1.84	1.12–1.78	0.21–1.34		0.16–2.5		1.01	0.9–1.1
Gd/mg kg^−1^	4.6–7.8	4.5–8.6	0.82–5.53		0.85–11		5.06	3.7–4.3
Tb/µg kg^*−1*^	690–1170	690–1180	140–950		130–1700		790	600–800
Dy/mg kg^−1^	4.0–6.9	4.1–6.6	0.65–4.89		0.84–9.1		4.53	3.9
Ho/µg kg^*−1*^	760–1310	790–1250	120–990		160–1800		920	830
Er/mg kg^−1^	1.9–3.6	2.2–3.6	0.32–3.16		0.46–4.9		2.67	2.3
Tm/mg kg^−1^	260–490	270–510	50–520		70–700		0.40	300
Yb/mg kg^−1^	2.0–3.3	1.8–3.4	0.32–3.27		0.42–4.5		2.58	1.56–2.36
Lu/ µg kg^*−*1^	280–500	300–540	40–500		60–620		390	260–360
Ta/µg kg^−1^	800–1200	700–1400	10–2064				1010	800–1000

^1^median values – Geochemical atlas of Europe (Salminen et al. 2006)

**Table 3:** Ranges of calculated local enrichment factors (LEFs) along with their respective threshold values estimated by the M2MAD and TIF approaches of each TCE in the two different regions (North and South) as well as threshold values for the combined dataset. All threshold values are given in mg kg^−1^ apart from In, Tb, Ho, and Ta where threshold values are given in µg kg^−1^ and therefore set in italic

	North			South			Combined	
	LEF	M2MAD	TIF	LEF	M2MAD	TIF	M2MAD	TIF
Sc	–	21.7	22.3	–	17.8	18.5	18.9	19.8
Y	0.6–1.4	34.2	35.1	0.8–1.3	29.8	31.0	32	34.8
Ga	0.8–1.6	25	24	0.9–1.5	20	24	20	24
Ge	0.9–2.0	2.5	2.5	0.8–1.5	2.2	2.2	2.4	2.4
Nb	0.8–1.9	15.3	15.6	0.6–1.7	16.8	19.4	16.1	17.1
*In*	*0.6–2.3*	*265*	*267*	*0.8–1.6*	*158*	*168*	*241*	*232*
La	0.7–1.0	43.9	45.4	0.8–1.5	54.3	56.4	47	51.5
Ce	0.6–1.2	82	90	0.7–1.5	112	114	91	100
Pr	0.7–1.3	10.7	11.3	0.7–1.5	13.9	13.9	11.5	12.6
Nd	0.7–1.3	38	41	0.7–1.5	51	50	42	47
Sm	0.8–1.4	7.9	8.1	0.7–1.5	9.3	9.9	8.8	9.2
Eu	0.8–1.4	1.83	1.9	0.8–1.3	1.69	1.95	1.79	1.92
Gd	0.8–1.3	8.3	8.4	0.8–1.4	8.3	8.7	8.3	8.6
*Tb*	*0.8–1.5*	*1240*	*1280*	*0.8–1.3*	*1160*	*1230*	*1250*	*1270*
Dy	0.8–1.5	7.7	7.8	0.8–1.3	6.6	7.0	7.2	7.5
*Ho*	0.8–1.5	*1450*	*1470*	*0.8–1.3*	*1280*	*1300*	*1400*	*1430*
Er	0.7–1.5	4.0	4.1	0.8–1.3	3.8	3.9	4	4.2
Tm	0.8–1.7	510	530	0.7–1.4	490	510	530	560
Yb	0.8–1.7	3.2	3.3	0.7–1.5	3.0	3.1	3.1	3.4
Lu	0.7–1.5	500	520	0.8–1.4	520	540	520	530
*Ta*	*0.7–2.0*	*1400*	*1400*	*0.7–1.8*	*1300*	*1400*	*1300*	*1400*

### Instrumentation and Measurement Procedures

All measurements were conducted using a tandem mass spectrometer with inductively coupled plasma (ICP-MS/MS) using an optimized method for the measurement of TCEs in sediment digests (Klein et al. 2021). The ICP-MS/MS system consists of an Agilent 8800 ICP-MS/MS (Agilent 8800, Agilent Technologies, Tokyo, Japan) coupled to an ESI SC-4 DX FAST autosampler (Elemental Scientific, Omaha, Nebraska, USA) equipped with an ultra-low penetration air (ULPA) filtration unit (Elemental Scientific, Omaha, Nebraska, USA). For each measurement, the instrument was optimized on a daily basis using a tune solution containing Li, Co, Y, Ce, and Tl (10 μg L^−1^). Optimal tuning and instrument parameters can be found in the supplemental information Table A.4. An external calibration, covering a concentration range from 0.1 μg L^−1^ to 100 μg L^−1^ for all analytes, was used to allow quantification of each element. Each solution for instrument tuning and quantification was prepared from custom-made multielement standards (Inorganic Ventures, Christiansburg, USA) on a daily basis. A solution containing 10 μg L^−1^ of Ir and Rh was dosed online to the sample solutions to allow for drift correction. To monitor potential carryover effects, wash blanks of 2% (*w*/*w*) HNO_3_ were measured after each sample triplicate.

### Data Processing and Calculations

Multielement data were pre-processed using MassHunter version 4.4 (Agilent Technologies, Tokyo, Japan) in combination with a custom-written Excel^©^ spreadsheet. This spreadsheet was used to calculate limits of detection (*LOD*) (3 × *SD*) as well as the limits of quantification (*LOQ*) (10 × *SD*) of the method using procedural blanks (*n* = 6) (MacDougall and Crummett 1980). Furthermore, combined uncertainties (*U*, *k* = 2) for each sample and reference material were calculated using a simplified Kragten approach following Reese et al. (2019) (Kragten 1994; Reese et al. 2019). The measurement precision of the samples and the reproducibility of multiple digests were taken into account.

The significant number of digits is given according to GUM and EURACHEM guidelines, whereby the highest uncertainty of all measured samples determines the significant number of digits to be presented with the values (Magnusson et al. 2015; Ellison and Williams 2012).

### Calculation of Local Enrichment Factors and Preliminary Reference Thresholds

For evaluation and estimation of the TCE load in North Sea sediments, both local enrichment factors (LEF) and preliminary reference thresholds were determined. Calculation of LEF is carried out according to Matys Grygar and Popelka (2016). The natural mass fraction (*X*_*GBF*_) of a target element *X* is determined by an empirical function of a suitable reference element (in this case Sc, *f(Sc)*). The LEF is then calculated according to the following formula for the respective elements (Matys Grygar et al. 2014; Matys Grygar and Popelka 2016).

$${\text{LEF}}_{{\text{X}}} = \frac{X}{{X_{\text{GBF}} }}{ } {\text{with }}X_{\text{GBF}} = f\left( \text{Sc} \right)$$

A list showing the respective function for each TCE for the calculation of *X*_*GBF*_ using Sc as reference element as well as the coefficient of determination (*r*^2^) of the chosen function can be found in the supplemental information Table A.5.

The preliminary reference thresholds are calculated following Reimann et al. (2018) using the M2MAD and TIF approach. According to Reimann et al. (2018), these two estimates for threshold values are calculated as follows:

$${\text{M}}2{\text{MAD}} = 10^{b}$$

$${\text{where }}b = {\text{median}}(\log_{10} \left( {X_{i} } \right)) + 2{\text{*MAD}}(\log_{10} \left( {X_{i} } \right))$$

$${\text{and MAD}} = 1.48{\text{ median}}\left( {x_{i} - {\text{median}}\left( {X_{i} } \right)} \right){ }$$

$${\text{TIF}} = {\text{Q}}3 + 1.5{\text{ IQR }}$$

*X*_*i*_ represents the mass fractions of the respective TCE and Q3 is the 3rd quartile of the dataset while IQR is the interquartile range (Q3 – Q1) (Reimann et al. 2018).

## Results and Discussion

### Mass Fractions of TCEs in North Sea Sediments

For this study, the < 20 µm grain size fraction of 50 sediment samples was analyzed. Sampling locations were either located close to the southern coast of Heligoland or further offshore in the North Sea.

Despite the comparatively small geographical area, the North Sea is a very unique and dynamic marine ecosystem with very different geological characteristics, which is partly due to intensive sediment movements in the coastal area, either by anthropogenic sediment relocation or by natural currents (Ducrotoy et al. 2000; Federal Maritime and Hydrographic Agency (BSH) 2016). The investigated part of the North Sea is considered to be anthropogenically affected, which becomes particularly evident within long-term monitoring data (Federal Maritime and Hydrographic Agency (BSH) 2016). Mass fractions of legacy pollutants like Zn, Pb, and Hg often exceed background and even low effect threshold values (*NOAA effect range low*). In many cases, elevated mass fractions are observed near the coast, which indicates possible discharges by rivers or other near-shore anthropogenic sources (Federal Maritime and Hydrographic Agency (BSH) 2009; Federal Maritime and Hydrographic Agency (BSH) 2016).

Additionally, the southern area of Heligoland is anthropogenically influenced by the relocation of sediment from the Hamburg harbor, but also by the input loads of the Elbe River. Until 2011 6.5 million tons of harbor sediments were relocated near to the sampling position HPAE3 (Federal Maritime and Hydrographic Agency (BSH) 2016; Hamburg Port Authority (HPA) 2021) (cf. Fig. 1). Therefore, anthropogenic inputs of TCEs via the river Elbe will most likely be measurable at this geographic location. For the other region, comparatively lower loads are to be expected. Nevertheless, diluted H_2_SO_4_ acid from the titanium oxide production containing various heavy metal impurities was deposited in this region until 1989, which may represent a further potential environmental pollution source (Federal Maritime and Hydrographic Agency (BSH) 2016; Pickaver 1982). Furthermore, it is suspected that the TCE load in sediments may rise within the next years or decades as different offshore wind farms were built in this region since 2012 (Federal Maritime and Hydrographic Agency (BSH) 2020; Kirchgeorg et al. 2018; Ramírez et al. 2021; Reese et al. 2020). Hereafter, the temporal variations of the TCEs (Ga, Ge, Nb, In, REEs, and Ta) are described and compared with values from the literature. Detailed information on measured TCE mass fractions for all stations can be found in the supplemental information Table A. 6.

#### Gallium

**Fig. 2:**
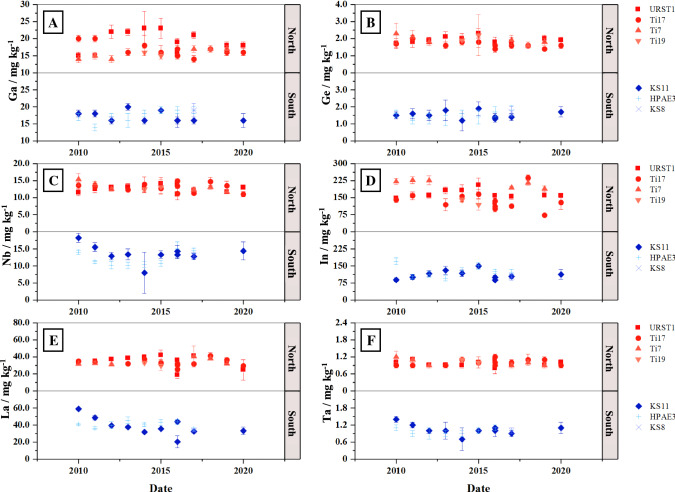
Elemental mass fraction in the < 20 µm grain size fraction of the two observed North Sea regions over the investigated time frame of ten years. All error bars correspond to expanded uncertainties (*U*(*k* = 2))

**Fig. 3:**
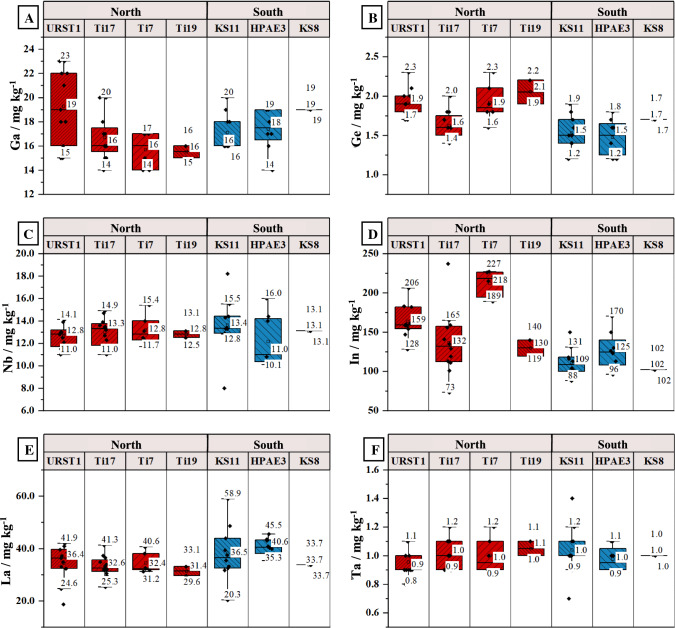
Variation of elemental mass fractions in the < 20 µm grain size fraction of TCEs within each observed sample location. Each box covers all measured mass fractions within the observed time frame of ten years. Numbers in the box plots indicate upper and lower whiskers as well as median values

Figure 2a shows the Ga mass fractions of the regions “North” and “South” over the observed time (2010–2020). For both regions, it can be seen that mass fractions show similar ranges between 14.0 mg kg^−1^ ± 1.0 mg kg^−1^ and 23 mg kg^−1^ ± 5 mg kg^−1^. Furthermore, it becomes evident that the variability of Ga mass fractions over time appears to be relatively small. The boxplots in Fig. 3a represent all measured Ga mass fractions of the time series at the given stations. These boxplots indicate that station URST1 shows the highest variability over the ten years ranging from 15 mg kg^−1^ ± 1 mg kg^−1^ to 23 mg kg^−1^ ± 5 mg kg^−1^. All other stations show Ga mass fractions ranging between 14 and 20 mg kg^−1^. Overall, a median value of 17 mg kg^−1^ ± 1 µg kg^−1^can be derived for both regions.

#### Germanium

The Ge mass fraction ranges between 1.2 ± 0.2 mg kg^−1^ and 2.3 mg kg^−1^ ± 0.6 mg kg^−1^ for all samples. Figure 2b shows only minor variability in Ge mass fractions for both regions over time. Referring to the boxplots in Fig. 3b, it is evident that stations of the North region feature slightly higher mass fractions compared to the South region (median_North_ = 1.8 µg kg^−1^; median_South_ = 1.5 µg kg^−1^). Furthermore, stations KS11 and HPAE3 (South region) feature the highest variability over the observed time frame.

#### Niobium

Nb mass fractions of the North region range from 11.0 µg kg^−1^ ± 0.8 µg kg^−1^ to 15.4 µg kg^−1^ ± 1.7 µg kg^−1^, whereas the South region shows a range from 8 µg kg^−1^ ± 6 µg kg^−1^ to 18.2 µg kg^−1^ ± 1.3 µg kg^−1^. Mass fractions of Nb show no significant changes over time in the North region. In contrast, the stations in the South region like KS11 show some variation between the years 2010 and 2014 (cf. Fig. 2c), thus resulting in a range of Nb mass fractions between 8 mg kg^−1^ ± 6 mg kg^−^^1^ and 18.2 mg kg^−1^ ± 1.3 mg kg^−1^ for this region. However, considering the respective uncertainties, these variabilities do not appear to be significant. Notwithstanding the region, boxplots in Fig. 3c show that all stations have similar variability and median mass fractions around 12.0 mg kg^−1^ ± 1.0 mg kg^−1^.

#### Indium

In mass fractions of the North region range from 73 µg kg^−1^ ± 4 µg kg^−1^ to 237 µg kg^−1^ ± 13 µg kg^−1^, whereas the South region shows a range from 88 µg kg^−1^ ± 7 µg kg^−1^ to 170 µg kg^−1^ ± 15 µg kg^−1^. Figure 2d shows In mass fractions of the two regions over time. It can be seen that the In mass fractions of stations within the North region vary noticeably from each other, but remain rather stable over the considered period of 10 years, whereas the stations of the South region are more homogeneous over time. This fact can also be seen in Fig. 3d. It becomes evident that station Ti7 shows the highest In mass fractions while Ti17 and Ti9 lay within the range of the South region.

#### Rare Earth Elements

All mass fractions of the rare earth elements (REEs) show similar to identical trends over the observed time. Therefore, only La is discussed in detail as a representative for all other REEs (cf. Table 1).

As displayed in Fig. 2e, the North region features La mass fractions ranging from 18.7 mg kg^−1^ ± 3.7 mg kg^−1^ to 41.9 mg kg^−1^ ± 6.1 mg kg^−1^. Station KS11 of the South region shows decreasing mass fractions in the years from 2010 to 2014 (58.9 mg kg^−1^ ± 1.6 mg kg^−1^ to 31.7 mg kg^−1^ ± 1.3 mg kg^−1^). After 2014, the mass fractions of station KS11 follow the course of the remaining two stations (HPAE3 and KS8) with La mass fractions between 33.7 mg kg^−1^ ± 1.6 mg kg^−1^ and 45.5 mg kg^−1^ ± 4.1 mg kg^−1^. Reese et al. (2019) also observed elevated mass fractions of the REEs La, Ce, Pr, Nd, and Sm in the Elbe estuary and attributed this to possible discharges of phosphogypsum in the Rhine delta (Reese et al. 2019). Due to the proximity of station KS11 to the Elbe estuary, a similar effect may be occurring here. The boxplot in Fig. 3e shows that even though the mass fractions of station KS11 show a higher variation over time, all stations of the South and North regions state similar median mass fractions ranging from 32.6 mg kg^−1^ to 40.6 mg kg^−1^. In addition, PAAS (post-archaean average shale) normalization of the REEs does not indicate any anomalies or anthropogenic influences (cf. supplemental information Fig. A.1 of two stations from both regions showing the PAAS-normalized REE patterns).

#### Tantalum

The Ta mass fraction ranges between 0.7 mg kg^−1^ ± 0.4 mg kg^−1^ and 1.4 mg kg^−1^ ± 0.1 mg kg^−1^ for all samples. Figure 2f shows the Ta mass fractions for all stations over the observed time of ten years. All stations of the South and North regions show a homogeneous trend as shown in Fig. 3f. The median mass fraction of both regions ranges from 0.9 mg kg^−1^ to 1.1 mg kg^−1^.

### Evaluation of Possible TCE Contamination in North Sea Sediments

In order to preliminary classify the determined TCE mass fractions in the North Sea, reference values from other studies were compared with the TCE mass fractions determined in this study. For an optimal comparison, data from a similar matrix and region would be preferable. However, TCE data from environmental samples are still scarce. Therefore, mass fraction ranges and median values from coastal, riverine, and lake sediment (Bačić et al. 2021; Dolor et al. 2009; Ohta et al. 2010; Salminen et al. 2006) as well as riparian soil (Lučić et al. 2021), and the respective crustal abundances (Rudnick and Gao 2003) were used. Overall the determined TCE mass fractions in the North Sea (cf. Table 2) appear to fit well with the already observed ranges and the respective crustal abundances (UCC). However, the minimum mass fractions of the TCEs in the North Sea are often higher than the respective sediment literature values for coastal sediments. Given the respective crustal abundances of TCEs, especially In and some REEs seem to exceed these abundances. Nevertheless, under consideration of the respective uncertainties and due to the circumstance that the observed maximum values only occur temporally, it may be assumed that they are not significantly higher than the given UCC values. Thus, significant anthropogenic inputs of TCEs are relatively unlikely.

Comparing the two observed regions (North and South), there are only minor differences in the observed TCE mass fractions. However, for many of the TCEs (Ga, Ge, Nb, In, Eu, Gd, Dy, Ho, Yb, and Ta) mass fractions of the North region are elevated. This may be due to the partly different geological characteristics (e.g., net sedimentation and grain size distribution) of the sediments in these two regions of the North Sea (Ducrotoy et al. 2000; Federal Maritime and Hydrographic Agency (BSH) 2016).

#### Calculation of Local Enrichment Factors of TCEs in North Sea Sediments

To better evaluate the TCE load of the observed regions, local enrichment factors (LEF) were calculated for each element with Sc as reference element as the REEs, in general, appeared to be anthropogenically unaffected (cf. PAAS-normalized plots in supplemental information Fig. A.1).

Following the enrichment factor (EF) thresholds defined by Birch (2017), Table 3 gives an overview of the calculated LEFs of each TCE (Birch 2017). For all observed stations, at any time little to no pollution (LEF < 1.5) occurred. Only the LEFs of Ga, Ge, Nb, In, and Ta show elevated LEFs of 1.5 to 2.5, thus indicating the possibility of slightly polluted sediments. Additionally, it becomes evident that LEFs for both regions do not differ within their observed ranges. Potential anthropogenic influences, such as the construction and commissioning of offshore wind turbines, could be indicated by slightly enriched In LEFs (Kirchgeorg et al. 2018; Reese et al. 2020). Nevertheless, other possible sources of pollution, such as the Elbe or the deposition of diluted H_2_SO_4_, are not considered to be significant input sources for TCEs into the German Bight.

#### Estimation of Preliminary Reference Thresholds

For both regions, TCEs showed only little variation in mass fractions over the observed time of ten years. Additionally, the calculated LEFs indicate slight to no TCE pollution within this time frame. Nevertheless, both regions are partly affected by anthropogenic influences, and hence a true background value cannot be determined. Therefore, measured mass fractions for the TCEs were used to estimate preliminary reference thresholds. Two different methods were used (M2MAD and TIF) whereby the M2MAD method provides more conservative limits than the more robust TIF method. Table 3 summarizes the calculated threshold values for both regions. Both approaches show consistent threshold values for each analyzed TCE, thus indicating that the presented thresholds are a good estimate for future studies. Furthermore, there are no substantial differences between the thresholds of the two regions and values were also determined using the entire dataset.

## Conclusion

Within this work, a time series of ten years of sediment samples at two different locations in the German North Sea were analyzed for their mass fractions of the TCEs Ga, Ge, Nb, In, REEs, and Ta to investigate the occurrence and temporal variation of these elements. The regional scale and period of 10 years of the analyzed samples enabled the determination of robust threshold values for the analyzed TCEs within the German Bight. Even though the data do not indicate significant anthropogenic inputs of TCEs into the German North Sea up to now, this is likely to change over the next years to decades. Exact knowledge of mass fractions of TCEs in sediment samples will be of great benefit to gain better insights into the behavior of these elements in the marine environment and for the determination of their origin, fate, and distribution within complex land-river-sea systems.

Therefore, the reference values determined in this study will be very valuable for future environmental studies investigating the input of TCEs into the environment. One of which may be emissions of TCEs from offshore wind farms in the German Bight, which play a key role in the ongoing energy transition in Germany.

## Supplementary Information

Below is the link to the electronic supplementary material.

Supplementary file1 (DOCX 89 kb)
