# Supplementary material for: Occurrence and Temporal Variation of Technology-Critical Elements in North Sea Sediments—A Determination of Preliminary Reference Values
Source: Arch Environ Contam Toxicol. 2022 Apr 26;82(4):481–92. doi: 10.1007/s00244-022-00929-4 (PMC9079029; doi:10.1007/s00244-022-00929-4)
Supplement: Supplementary file 1 — Supplementary file1 (DOCX 89 kb) [file 244_2022_929_MOESM1_ESM.docx]

**Occurrence and temporal variation of technology-critical elements in North Sea sediments - a determination of preliminary reference values**

*Ole Klein^a,b^, Tristan Zimmermann^a^, Anna Ebeling^a,b^, Madita Kruse^c^,^,^Torben Kirchgeorg^d^ and Daniel Pröfrock^a^ **

^a^ Helmholtz-Zentrum Hereon, Institute of Coastal Environmental Chemistry, Inorganic Environmental Chemistry, Max-Planck Str. 1, 21502 Geesthacht, Germany

^b^ Universität Hamburg, Department of Chemistry, Inorganic and Applied Chemistry, Martin-Luther-King-Platz 6, 20146 Hamburg, Germany

^c^ HTWG Hochschule Konstanz, Department Mechanical Engineering, Alfred-Wachtel-Straße 8, 78462 Konstanz, Germany

^d.^ Bundesamt für Seeschifffahrt und Hydrographie (BSH), Marine Sciences Department, Marine Chemistry Laboratory – Shipping and Environment, Marine Sediments Section, Wüstland 2, 22589 Hamburg, Germany

*Corresponding author: daniel.proefrock@hereon.de

### **Supplemental information**

Table A.1 Sampling information including coordinates and sampling dates of sampled stations.

| **Campaign code** | **Station name** | **Latitude** | **Longitude** | **Sampling date** | **Assigned area** |
| --- | --- | --- | --- | --- | --- |
| **CE10001** | Ti17 | 54.33 | 7.71 | 22.01.2010 | North |
| **CE11002** | Ti17 | 54.34 | 7.70 | 19.01.2011 | North |
| **PE364** | Ti17 | 54.33 | 7.71 | 25.01.2013 | North |
| **PE385** | Ti17 | 54.33 | 7.71 | 31.01.2014 | North |
| **CE15001** | Ti17 | 54.33 | 7.71 | 01.02.2015 | North |
| **CE16011b** | Ti17 | 54.33 | 7.71 | 03.09.2016 | North |
| **LP20160725** | Ti17 | 54.34 | 7.73 | 25.07.2016 | North |
| **LP20160725** | Ti17 | 54.36 | 7.71 | 25.07.2016 | North |
| **CE17001** | Ti17 | 54.33 | 7.71 | 21.01.2017 | North |
| **AT261** | Ti17 | 54.36 | 7.71 | 12.04.2018 | North |
| **AT275** | Ti17 | 54.34 | 7.73 | 06.03.2019 | North |
| **LP20200629** | Ti17 | 54.34 | 7.73 | 29.06.2020 | North |
| **PE385** | Ti19 | 54.33 | 7.58 | 31.01.2014 | North |
| **CE15001** | Ti19 | 54.33 | 7.58 | 01.02.2015 | North |
| **CE10001** | Ti7 | 54.42 | 7.71 | 22.01.2010 | North |
| **CE11002** | Ti7 | 54.42 | 7.71 | 19.01.2011 | North |
| **CE12002** | Ti7 | 54.42 | 7.71 | 19.01.2012 | North |
| **AT248** | Ti7 | 54.42 | 7.71 | 31.05.2017 | North |
| **AT261** | Ti7 | 54.41 | 771 | 12.04.2018 | North |
| **AT275** | Ti7 | 54.42 | 7.71 | 06.03.2019 | North |
| **CE10001** | URST1 | 54.42 | 7.58 | 22.01.2010 | North |
| **CE11002** | URST1 | 54.42 | 7.58 | 19.01.2011 | North |
| **CE12002** | URST1 | 54.42 | 7.58 | 19.01.2012 | North |
| **PE364** | URST1 | 54.42 | 7.58 | 25.01.2013 | North |
| **PE385** | URST1 | 54.42 | 7.58 | 01.02.2014 | North |
| **CE15001** | URST1 | 54.42 | 7.58 | 01.02.2015 | North |
| **CE16011b** | URST1 | 54.42 | 7.58 | 03.09.2016 | North |
| **LP20160725** | URST1 | 54.43 | 7.57 | 28.07.2016 | North |
| **CE17001** | URST1 | 54.42 | 7.58 | 21.01.2017 | North |
| **AT275** | URST1 | 54.44 | 7.57 | 06.03.2019 | North |
| **LP20200629** | URST1 | 54.44 | 7.57 | 29.06.2020 | North |
| **CE10001** | HPAE3 | 54.05 | 7.97 | 22.01.2010 | South |
| **CE11002** | HPAE3 | 54.05 | 7.97 | 18.01.2011 | South |
| **CE12002** | HPAE3 | 54.05 | 7.97 | 18.01.2012 | South |
| **PE364** | HPAE3 | 54.05 | 7.97 | 24.01.2013 | South |
| **PE385** | HPAE3 | 54.05 | 7.97 | 30.01.2014 | South |
| **CE15001** | HPAE3 | 54.05 | 7.97 | 31.01.2015 | South |
| **CE16011b** | HPAE3 | 54.05 | 7.97 | 04.09.2016 | South |
| **CE17001** | HPAE3 | 54.05 | 7.97 | 20.01.2017 | South |
| **AT246** | KS8 | 54.03 | 8.21 | 13.03.2017 | South |
| **CE10001** | KS11 | 54.07 | 8.12 | 21.01.2010 | South |
| **CE11002** | KS11 | 54.06 | 8.13 | 18.01.2011 | South |
| **CE12002** | KS11 | 54.07 | 8.13 | 18.01.2012 | South |
| **PE364** | KS11 | 54.07 | 8.12 | 24.01.2013 | South |
| **PE385** | KS11 | 54.07 | 8.12 | 30.01.2014 | South |
| **CE15001** | KS11 | 54.07 | 8.13 | 31.01.2015 | South |
| **LP20160725** | KS11 | 54.07 | 8.10 | 25.07.2016 | South |
| **CE16011b** | KS11 | 54.07 | 8.12 | 04.09.2016 | South |
| **CE17001** | KS11 | 54.07 | 8.12 | 20.01.2017 | South |
| **AT246** | KS11 | 54.07 | 8.13 | 13.03.2017 | South |
| **LP20200629** | KS11 | 54.07 | 8.13 | 29.06.2020 | South |

Table A.2 List of measured analytes and their respective measuring mode. LODs and LOQs are given as range over a total of five measuring batches.

| **Analyte** | ***m*/*z* Q1** | ***m*/*z* Q2** | **Cell gas mode** | ***LOD* / µg L^-1^** | ***LOQ* / µg L^-1^** |
| --- | --- | --- | --- | --- | --- |
| **Sc** | 45 | 61 | N_2_O | 0.0028 - 0.029 | 0.008 - 0.09 |
| **Ga** | 71 |  | He | 0.006 - 0.07 | 0.022 - 0.24 |
| **Ge** | 72 | 88 | N_2_O | 0.007 - 0.06 | 0.024 - 0.2 |
| **Nb** | 93 | 125 | N_2_O | 0.016 - 0.16 | 0.05 - 0.5 |
| **In** | 115 | 115 | N_2_O | 0.01 - 0.02 | 0.015 - 0.04 |
| **La** | 139 | 155 | N_2_O | 0.008 - 0.04 | 0.027 - 0.15 |
| **Ce** | 140 | 156 | N_2_O | 0.007 - 0.09 | 0.023 - 0.3 |
| **Pr** | 141 | 157 | N_2_O | 0.0012 - 0.015 | 0.004 - 0.05 |
| **Nd** | 146 | 162 | N_2_O | 0.005 - 0.04 | 0.015 - 0.15 |
| **Sm** | 147 | 163 | N_2_O | 0.0014 - 0.012 | 0.005 - 0.04 |
| **Eu** | 153 | 169 | N_2_O | 0.00025 - 0.006 | 0.0008 - 0.02 |
| **Gd** | 157 | 173 | N_2_O | 0.0014 - 0.01 | 0.004 - 0.03 |
| **Tb** | 159 | 175 | N_2_O | 0.0003 - 0.005 | 0.001 - 0.017 |
| **Dy** | 163 | 179 | N_2_O | 0.001 - 0.012 | 0.003 - 0.04 |
| **Ho** | 165 | 181 | N_2_O | 0.00024 - 0.006 | 0.0006 - 0.02 |
| **Er** | 166 | 182 | N_2_O | 0.0005 - 0.008 | 0.0014 - 0.026 |
| **Tm** | 169 | 185 | N_2_O | 0.00005 - 0.004 | 0.00014 - 0.012 |
| **Yb** | 172 | 188 | N_2_O | 0.0012 - 0.019 | 0.004 - 0.06 |
| **Lu** | 179 | 191 | N_2_O | 0.00011 - 0.005 | 0.0003 - 0.015 |
| **Ta** | 181 | 197 | N_2_O | 0.014 - 2.2 | 0.05 - 7 |

Table A.3 Measured and certified mass fractions (mg kg^-1^) of the analyzed CRMs (GBW 07313, BCR-2 and GBW 07311). All given errors correspond to expanded uncertainties (U, k=2). All italic values are either information values provided on the certificate or from references a, b and c.(Balla et al., 2004; Jochum et al., 2016; Kirchenbaur et al., 2018).

|  | GBW 07313 (*n* = 10) | | | | | | | BCR-2 (*n* = 10) | | | | | | |  | GBW 07311 (*n* = 10) | | | | | |
| --- | --- | --- | --- | --- | --- | --- | --- | --- | --- | --- | --- | --- | --- | --- | --- | --- | --- | --- | --- | --- | --- |
|  | Certified value / mg kg^-1^ | | | Measured value / mg kg^-1^ | | | Recovery | Certified value / mg kg^-1^ | | | Measured value / mg kg^-1^ | | | Recovery | Certified value / mg kg^-1^ | | | Measured value / mg kg^-1^ | | | Recovery |
| **Sc** | 25.6 | ± | 2.9 | 26 | ± | 8 | 102% | 33 | ± | 2 | 37 | ± | 11 | 112% | 7.4 | ± | 0.4 | 6.8 | ± | 2.5 | 92% |
| **Ga** | 23.7 | ± | 1.7 | 24 | ± | 5 | 101% | 23 | ± | 2 | 21 | ± | 4 | 91% | 18.5 | ± | 0.9 | 18.3 | ± | 3.7 | 99% |
| **Ge** |  |  |  | 2.2 | ± | 0.7 | - | *1.46* | *±* | *0.26^a^* | 1.46 | ± | 0.3 | 100% | 1.81 | ± | 0.21 | 1.8 | ± | 0.4 | 99% |
| **Y** | 104 | ± | 5 | 105 | ± | 24 | 101% | 37 | ± | 2 | 34 | ± | 6 | 92% | 43 | ± | 5 | 29 | ± | 7 | 67% |
| **Nb** | *15.1* |  |  | 10.2 | ± | 2.0 | 68% | *12.4* | *±* | *0.02^a^* | 12 | ± | 4 | 97% | 25 | ± | 3 | 23 | ± | 6 | 92% |
| **In** |  |  |  | 0.169 | ± | 0.038 | - | 0.0913 | ± | 0.0009^b^ | 0.095 | ± | 0.012 | 104% | 1.9 | ± | 0.3 | 2.00 | ± | 0.32 | 105% |
| **La** | 67.8 | ± | 2.9 | 72 | ± | 15 | 106% | 25 | ± | 1 | 25.8 | ± | 3.8 | 103% | 30 | ± | 2 | 28 | ± | 5 | 93% |
| **Ce** | 92 | ± | 8 | 94 | ± | 17 | 102% | 53 | ± | 2 | 53 | ± | 6 | 100% | 58 | ± | 4 | 58 | ± | 8 | 100% |
| **Pr** | 20.1 | ± | 1.9 | 21 | ± | 5 | 104% | 6.8 | ± | 0.3 | 7.3 | ± | 1.2 | 107% | 7.4 | ± | 0.5 | 7.5 | ± | 1.1 | 101% |
| **Nd** | 91.8 | ± | 3.9 | 91 | ± | 19 | 99% | 28 | ± | 2 | 30 | ± | 5 | 107% | 27 | ± | 2 | 27 | ± | 4 | 100% |
| **Sm** | 21.5 | ± | 1.3 | 22 | ± | 4 | 102% | 6.7 | ± | 0.3 | 6.9 | ± | 1.2 | 103% | 6.2 | ± | 0.3 | 6.3 | ± | 1.0 | 102% |
| **Eu** | 5.3 | ± | 0.3 | 5.6 | ± | 1.4 | 106% | 2 | ± | 0.1 | 2.07 | ± | 0.38 | 104% | 0.6 | ± | 0.06 | 0.62 | ± | 0.14 | 103% |
| **Gd** | 22 | ± | 1.2 | 23 | ± | 5 | 105% | 6.8 | ± | 0.3 | 7.0 | ± | 1.0 | 103% | 5.9 | ± | 0.4 | 5.7 | ± | 0.9 | 97% |
| **Tb** | 3.4 | ± | 0.3 | 3.5 | ± | 0.8 | 103% | 0.54 | ± | 0.04 | 1.09 | ± | 0.15 | 202% | 1.13 | ± | 0.09 | 0.98 | ± | 0.17 | 87% |
| **Dy** | 19.9 | ± | 1.8 | 22 | ± | 5 | 111% |  |  |  | 6.8 | ± | 0.8 | - | 7.2 | ± | 0.6 | 6.2 | ± | 1.1 | 86% |
| **Ho** | 4.3 | ± | 0.2 | 4.3 | ± | 1.1 | 100% | 1.33 | ± | 0.06 | 1.37 | ± | 0.16 | 103% | 1.4 | ± | 0.2 | 1.22 | ± | 0.21 | 87% |
| **Er** | 11 | ± | 0.7 | 11.8 | ± | 3.2 | 107% |  |  |  | 3.9 | ± | 0.4 | - | 4.6 | ± | 0.5 | 3.6 | ± | 0.8 | 78% |
| **Tm** | 1.54 | ± | 0.14 | 1.42 | ± | 0.25 | 92% | 0.54 | ± | 0 | 0.54 | ± | 0.06 | 100% | 0.74 | ± | 0.09 | 0.56 | ± | 0.13 | 76% |
| **Yb** | 9.8 | ± | 1.1 | 9.5 | ± | 1.7 | 97% | 3.5 | ± | 0.2 | 3.8 | ± | 1.0 | 109% | 5.1 | ± | 0.6 | 4.1 | ± | 1.2 | 80% |
| **Lu** | 1.46 | ± | 0.19 | 1.6 | ± | 0.5 | 110% | 0.51 | ± | 0.02 | 0.52 | ± | 0.07 | 102% | 0.78 | ± | 0.06 | 0.59 | ± | 0.11 | 76% |
| **Ta** | *1.11* | *±* | *0.13^c^* | 0.83 | ± | 0.15 | 75% | *0.785* | *±* | *0.018^a^* | 0.78 | ± | 0.12 | 99% | 5.7 | ± | 0.5 | 5.2 | ± | 1.4 | 91% |

Table A.4 ICP-MS/MS instrument and plasma parameters.

| **Instrument configurations and settings** | | | | | |
| --- | --- | --- | --- | --- | --- |
| Sample introduction | double-pass quartz glass spray chamber | | | | |
| Nebulizer | self-aspirating MicroFlow (ESI) | | | | |
| Spray chamber Temperature | 2°C | | | | |
| Interface cones | Nickel | | | | |
| RF Power | 1550 W | | | | |
| RF Matching | 1.80 V | | | | |
| Used cell gases | He, N_2_O, H_2_ | | | | |
| **Lens Parameters** | **No Gas** | **He** | **N_2_O** | **H_2_** | **H_2_ HMI** |
| Extract Lens 1 | 0.0 V | -2.5 V | 0.0 V | 0 V | 0.0 V |
| Extract Lens 2 | -170 V | -195 V | -155 V | -150 V | -195 V |
| Omega Bias | -100 V | -105 V | -95 V | -80 V | -105 V |
| Omega Lens | 8.9 V | -11.4 V | 9.4 V | 10.1 V | 10.0 V |
| Q1 Entrance | 0 V | 1 V | 0 V | 1 V | -4 V |
| Q1 Exit | -3 V | 3 V | -19 V | -2 V | 0 V |
| Cell Focus | 1.0 V | 1.0 V | 10.0 V | 0.0 V | 2.0 V |
| Cell Entrance | -40 V | -50 V | -50 V | -50 V | -50 V |
| Cell Exit | -50 V | -60 V | -60 V | -60 V | -60 V |
| Deflect | 14.4 v | -3.0 V | 10.0 V | 4.2 V | -4.2 V |
| Plate Bias | -50 V | -60 V | -60 V | -60 V | -60 V |
| **Cell parameters** |  |  |  |  |  |
| Cell gas flow | none | 4.5 mL min^-1^ | 20% | 6.0 mL min^-1^ | 6.0 mL min^-1^ |
| OctP Bias | -8.0 V | -20 V | -0.5 V | -18.0 V | -18.0 V |
| OctP RF | 130 V | 200 V | 200 V | 200 V | 170 V |
| Energy Discrimination | 5.0 V | 5.0 V | -5.0 V | 0.0 V | 0.0 V |

Table A.5 Functions to calculate the respective M_GBF_ of each TCE along with the achieved r² of the applied function.

|  | **North** | | **South** | |
| --- | --- | --- | --- | --- |
| **Element** | **M_GBF_** | **r²** | **M_GBF_** | **r²** |
| Y | 16.5 ∙ Sc | 0.99 | 16.6 ∙ Sc | 0.98 |
| Ga | 12.1 ∙ Sc | 0.99 | 12 ∙ Sc | 0.98 |
| Ge | 1.1 ∙ Sc | 0.97 | 1.2 ∙ Sc | 0.97 |
| Nb | 9.0 ∙ Sc | 0.96 | 8.8 ∙ Sc | 0.98 |
| In | 81.4 ∙ Sc | 0.96 | 112.4 ∙ Sc | 0.97 |
| La | 27.5 ∙ Sc | 0.99 | 23.5 ∙ Sc | 0.99 |
| Ce | 55.3 ∙ Sc | 0.99 | 48.5 ∙ Sc | 0.99 |
| Pr | 6.9 ∙ Sc | 0.99 | 6.1 ∙ Sc | 1.00 |
| Nd | 25.2 ∙ Sc | 0.99 | 22.2 ∙ Sc | 1.00 |
| Sm | 4.9 ∙ Sc | 0.99 | 4.6 ∙ Sc | 1.00 |
| Eu | 1.0 ∙ Sc | 1.00 | 1 ∙ Sc | 1.00 |
| Gd | 4.3 ∙ Sc | 0.99 | 4.3 ∙ Sc | 1.00 |
| Tb | 619.5 ∙ Sc | 1.00 | 639.6 ∙ Sc | 1.00 |
| Dy | 3.6 ∙ Sc | 1.00 | 3.8 ∙ Sc | 1.00 |
| Ho | 682.8 ∙ Sc | 1.00 | 716.3 ∙ Sc | 1.00 |
| Er | 1.9 ∙ Sc | 1.00 | 2 ∙ Sc | 0.99 |
| Tm | 247.7 ∙ Sc | 0.96 | 263.9 ∙ Sc | 0.98 |
| Yb | 1.6 ∙ Sc | 0.97 | 1.8 ∙ Sc | 0.99 |
| Lu | 268 ∙ Sc | 1.00 | 262.4 ∙ Sc | 1.00 |
| Ta | 704.1 ∙ Sc | 0.98 | 684.7 ∙ Sc | 0.95 |

Table A.6 Mass fractions (mg kg^-1^ or µg kg^-1^) of the size fraction <20 µm and the percentages of the <20 µm fraction of each sample. All given errors correspond to expanded uncertainties (U(k=2)).

|  | **Sc/mg kg^-1^** | **Ga/mg kg^-1^** | **Ge/mg kg^-1^** | **Y/mg kg^-1^** | **Nb/mg kg^-1^** | **In/µg kg^-1^** | **La/mg kg^-1^** | **Ce/mg kg^-1^** | **Pr/mg kg^-1^** | **Nd/mg kg^-1^** | **Sm/mg kg^-1^** |
| --- | --- | --- | --- | --- | --- | --- | --- | --- | --- | --- | --- |
| URST1_2010 | 11.9 ± 0.3 | 15.0 ± 1.0 | 1.7 ± 0.3 | 23.0 ± 1.0 | 11.5 ± 0.9 | 147 ± 9 | 33.0 ± 0.9 | 68.0 ± 2.0 | 8.3 ± 0.2 | 31.0 ± 1.0 | 6.3 ± 0.3 |
| URST1_2011 | 12.5 ± 0.5 | 15.0 ± 1.0 | 1.8 ± 0.3 | 23.1 ± 1.1 | 12.5 ± 1.0 | 158 ± 17 | 34.9 ± 1.7 | 72 ± 4 | 8.8 ± 0.4 | 32.0 ± 1.0 | 6.5 ± 0.4 |
| URST1_2012 | 17.8 ± 0.9 | 22.0± 2.0 | 1.9 ± 0.2 | 25.9 ± 1.3 | 13.0 ± 0.7 | 160 ± 11 | 37.2 ± 1.1 | 74.0 ± 2.0 | 9.3 ± 0.2 | 34.0 ± 1.0 | 6.8 ± 0.2 |
| URST1_2013 | 17.8 ± 1.1 | 22.0 ± 1.0 | 2.1 ± 0.3 | 27.0 ± 0.5 | 13.2 ± 0.9 | 182 ± 15 | 38.5 ± 0.8 | 77.0 ± 2.0 | 9.5 ± 0.1 | 35.0 ± 1.0 | 7.0 ± 0.3 |
| URST1_2014 | 17.2 ± 1.6 | 23 ± 5 | 2.0 ± 0.3 | 27.8 ± 2.1 | 12.8 ± 1.4 | 183 ± 23 | 39.6 ± 2.7 | 80 ± 6 | 9.9 ± 0.7 | 37.0 ± 2.0 | 7.4 ± 0.6 |
| URST1_2015 | 18.1 ± 2.8 | 23.0 ± 3.0 | 2.3 ± 0.3 | 30.3 ± 4.6 | 14.1 ± 1.9 | 206 ± 31 | 41.9 ± 6.1 | 84 ± 12 | 10.6 ± 1.5 | 39 ± 5 | 8.0 ± 1.1 |
| URST1_2016 | 8.1 ± 0.9 | 16.0 ± 1.0 | 1.7 ± 0.2 | 11.9 ± 0.8 | 13.9 ± 0.4 | 128 ± 7 | 18.7 ± 3.7 | 45 ± 6 | 6.3 ± 0.8 | 24.0 ± 2.0 | 5.2 ± 0.2 |
| URST1_2016 | 8.1 ± 0.9 | 16.0 ± 1.0 | 1.7 ± 0.2 | 11.9 ± 0.8 | 13.9 ± 0.4 | 128 ± 7 | 18.7 ± 3.7 | 45 ± 6 | 6.3 ± 0.8 | 24.0 ± 2.0 | 5.2 ± 0.2 |
| URST1_2017 | 16.9 ± 1.0 | 21.0 ± 1.0 | 1.8 ± 0.3 | 27.4 ± 0.9 | 12.1 ± 1.0 | 154 ± 12 | 40.9 ± 2.3 | 82.0 ± 3.0 | 10.3 ± 0.4 | 37.0 ± 1.0 | 7.5 ± 0.3 |
| URST1_2019 | 15.2 ± 0.4 | 18.0 ± 1.0 | 2.0 ± 0.2 | 22.1 ± 0.8 | 11.7 ± 0.6 | 161 ± 9 | 32.3 ± 1.1 | 65.0 ± 3.0 | 8.3 ± 0.2 | 31.0 ± 1.0 | 6.3 ± 0.2 |
| URST1_2020 | 13.9 ± 4.0 | 18.0 ± 1.0 | 1.9 ± 0.2 | 14.5 ± 0.6 | 13.0 ± 0.6 | 159 ± 9 | 25 ± 12 | 49.0 ± 19 | 6.7 ± 2.2 | 25 ± 7 | 5.6 ± 1.4 |
| Ti17_2010 | 16.9 ± 0.8 | 20.0 ± 1.0 | 1.7 ± 0.2 | 25.4 ± 1.3 | 13.6 ± 0.9 | 141 ± 13 | 34.9 ± 1.5 | 73.0 ± 3.0 | 8.9 ± 0.3 | 32.0 ± 2.0 | 6.5 ± 0.3 |
| Ti17_2011 | 16.4 ± 0.5 | 20.0 ± 1.0 | 2.0 ± 0.2 | 24.6 ± 0.8 | 13.2 ± 0.9 | 159 ± 9 | 33.9 ± 1.4 | 69.0 ± 3.0 | 8.5 ± 0.4 | 31.0 ± 2.0 | 6.3 ± 0.3 |
| Ti17_2013 | 14.2 ± 0.5 | 16.0 ± 1.0 | 1.6 ± 0.2 | 22.6 ± 1.5 | 12.3 ± 0.3 | 119 ± 26 | 32.0 ± 1.7 | 66 ± 4 | 8.1 ± 0.5 | 29.0 ± 2.0 | 5.9 ± 0.5 |
| Ti17_2014 | 16.4 ± 2.7 | 18.0 ± 3.0 | 1.8 ± 0.2 | 28.0 ± 4.3 | 13.9 ± 2.2 | 156 ± 22 | 37.3 ± 5.5 | 79 ± 11 | 9.6 ± 1.4 | 35 ± 5 | 7.2 ± 1.0 |
| Ti17_2015 | 14.1 ± 1.5 | 16.0 ± 2.0 | 1.8 ± 0.3 | 25.9 ± 2.9 | 12.7 ± 1.5 | 165 ± 20 | 33.1 ± 3.3 | 69 ± 7 | 8.5 ± 0.8 | 31.0 ± 3.0 | 6.6 ± 0.7 |
| Ti17_2016 | 9.0 ± 2.7 | 17.0 ± 1.0 | 1.4 ± 0.2 | 13.3 ± 0.7 | 14.9 ± 0.6 | 101 ± 14 | 25.3 ± 1.5 | 58 ± 4 | 7.8 ± 0.3 | 29.0 ± 1.0 | 6.0 ± 0.2 |
| Ti17_2016 | 9.0± 2.7 | 17.0 ± 1.0 | 1.4 ± 0.2 | 13.3 ± 0.7 | 14.9 ± 0.6 | 101 ± 14 | 25.3 ± 1.5 | 58 ± 4 | 7.8 ± 0.3 | 29.0 ± 1.0 | 6.0 ± 0.2 |
| Ti17_2016 | 9.0 ± 2.7 | 17.0 ± 1.0 | 1.4 ± 0.2 | 13.3 ± 0.7 | 14.9 ± 0.6 | 101 ± 14 | 25.3 ± 1.5 | 58 ± 4 | 7.8 ± 0.3 | 29.0 ± 1.0 | 6.0 ± 0.2 |
| Ti17_2017 | 12.8 ± 0.5 | 14.0 ± 1.0 | 1.6 ± 0.2 | 22.1 ± 1.7 | 11.3 ± 0.5 | 113 ± 4 | 31.8 ± 1.2 | 66.0 ± 3.0 | 8.1 ± 0.3 | 30.0 ± 1.0 | 6.1 ± 0.4 |
| Ti17_2018 | 15.5 ± 1.3 | 17.0 ± 1.0 | 1.6 ± 0.2 | 27.8 ± 0.7 | 14.7 ± 1.3 | 237 ± 13 | 41.3 ± 3.6 | 88 ± 7 | 11.0 ± 1.0 | 40.0 ± 4.0 | 8.6 ± 0.9 |
| Ti17_2019 | 15.3 ± 1.7 | 16.0 ± 1.0 | 1.4 ± 0.1 | 20.1 ± 0.8 | 13.5 ± 1.4 | 73 ± 4 | 36.5 ± 2.8 | 72 ± 5 | 9.2 ± 0.5 | 33.0 ± 2.0 | 6.7 ± 0.2 |
| Ti17_2020 | 13.4 ± 0.2 | 16.0 ± 1.0 | 1.6 ± 0.2 | 17.3 ± 0.8 | 11.0 ± 0.8 | 129 ± 30 | 29.5 ± 0.9 | 59.0 ± 1.0 | 7.6 ± 0.2 | 27.0 ± 1.0 | 5.5 ± 0.2 |
| Ti7_2010 | 12.5 ± 0.3 | 14.0 ± 1.0 | 2.3 ± 0.6 | 28.1 ± 1.3 | 15.4 ± 1.7 | 221 ± 13 | 31.8 ± 0.9 | 68.0 ± 1.0 | 8.2 ± 0.2 | 30.0 ± 1.0 | 6.7 ± 0.3 |
| Ti7_2011 | 12.1 ± 0.5 | 15.0 ± 1.0 | 2.1 ± 0.4 | 27.9 ± 1.4 | 14.0 ± 0.8 | 227 ± 16 | 32.6 ± 1.5 | 69.0 ± 3.0 | 8.5 ± 0.3 | 32.0 ± 1.0 | 6.9 ± 0.4 |
| Ti7_2012 | 12.2 ± 0.4 | 14.0 ± 1.0 | 1.8 ± 0.2 | 27.1 ± 1.1 | 12.5 ± 0.6 | 226 ± 20 | 31.2 ± 1.3 | 67.0 ± 3.0 | 8.2 ± 0.4 | 31.0 ± 2.0 | 6.7 ± 0.4 |
| Ti7_2017 | 18.0 ± 6.1 | 17.0 ± 1.0 | 1.9 ± 0.3 | 31.5 ± 9.1 | 12.3 ± 0.5 | 194 ± 6 | 41 ± 12 | 82 ± 21 | 10.2 ± 2.3 | 38 ± 9 | 8.2 ± 1.6 |
| Ti7_2018 | 14.9 ± 0.7 | 17.0 ± 1.0 | 1.6 ± 0.2 | 25.0 ± 0.9 | 13.1 ± 0.6 | 215 ± 10 | 38.1 ± 1.1 | 77.0 ± 3.0 | 9.9 ± 0.3 | 36.0 ± 1.0 | 7.6 ± 0.2 |
| Ti7_2019 | 14.6 ± 0.3 | 17.0 ± 1.0 | 1.8 ± 0.2 | 25.8 ± 1.6 | 11.7 ± 0.3 | 189 ± 12 | 32.1 ± 0.5 | 66.0 ± 2.0 | 8.4 ± 0.2 | 31.0 ± 1.0 | 6.7 ± 0.2 |
| Ti19_2014 | 12.8 ± 0.7 | 16.0 ± 1.0 | 1.9 ± 0.3 | 22.5 ± 1.9 | 12.5 ± 0.5 | 140 ± 10 | 33.1 ± 1.6 | 70.0 ± 3.0 | 8.5 ± 0.4 | 31.0 ± 2.0 | 6.3 ± 0.3 |
| Ti19_2015 | 12.2 ± 1.5 | 15.0 ± 1.0 | 2.2 ± 1.2 | 21.9 ± 0.9 | 13.1 ± 2.2 | 119 ± 23 | 29.6 ± 6.1 | 62 ± 10 | 7.4 ± 2.1 | 26 ± 9 | 5.3 ± 2.0 |
| KS11_2010 | 14.5 ± 1.2 | 18.0 ± 1.0 | 1.5 ± 0.2 | 31.6 ± 1.1 | 18.2 ± 1.3 | 89 ± 8 | 58.9 ± 1.6 | 120.0 ± 3.0 | 14.9 ± 0.3 | 54.0 ± 1.0 | 10.2 ± 0.3 |
| KS11_2011 | 14.4 ± 1.1 | 18.0 ± 1.0 | 1.6 ± 0.3 | 27.2 ± 1.0 | 15.5 ± 1.3 | 101 ± 12 | 48.6 ± 2.5 | 97.0 ± 3.0 | 12.1 ± 0.4 | 44.0 ± 1.0 | 8.4 ± 0.4 |
| KS11_2012 | 13.2 ± 0.4 | 16.0 ± 1.0 | 1.5 ± 0.3 | 22.0 ± 1.1 | 12.9 ± 1.1 | 116 ± 14 | 39.4 ± 1.8 | 80 ± 4 | 10.0 ± 0.5 | 36.0 ± 2.0 | 7.1 ± 0.3 |
| KS11_2013 | 18 ± 7 | 20.0 ± 1.0 | 1.8 ± 0.6 | 23.6 ± 1.0 | 13.4 ± 1.6 | 131 ± 18 | 37.5 ± 1.2 | 73.0 ± 3.0 | 9.1 ± 0.3 | 33.0 ± 2.0 | 6.5 ± 0.4 |
| KS11_2014 | 13.8 ± 0.6 | 16.0 ± 1.0 | 1.2 ± 0.6 | 19.8 ± 0.8 | 8 ± 6 | 118 ± 14 | 31.7 ± 1.3 | 62.0 ± 2.0 | 8.1 ± 0.4 | 30.0 ± 3.0 | 6.0 ± 0.5 |
| KS11_2015 | 15.6 ± 1.9 | 19.0 ± 1.0 | 1.9 ± 0.4 | 22.1 ± 0.7 | 13.3 ± 1.1 | 150 ± 9 | 35.5 ± 1.2 | 70.0 ± 2.0 | 8.7 ± 0.2 | 32.0 ± 1.0 | 6.2 ± 0.3 |
| KS11_2016 | 8.8 ± 1.6 | 16.0 ± 2.0 | 1.4 ± 0.2 | 14.2 ± 1.8 | 13.3 ± 1.2 | 88 ± 7 | 20.3 ± 7.1 | 46 ± 12 | 6.5 ± 1.4 | 25 ± 5 | 5.3 ± 0.8 |
| KS11_2016 | 8.8 ± 1.6 | 16.0 ± 2.0 | 1.4 ± 0.2 | 14.2 ± 1.8 | 13.3 ± 1.2 | 88 ± 7 | 20.3 ± 7.1 | 46 ± 12 | 6.5 ± 1.4 | 25 ± 5 | 5.3 ± 0.8 |
| KS11_2017 | 13.7 ± 0.5 | 16.0 ± 1.0 | 1.4 ± 0.2 | 20.8 ± 1.0 | 12.8 ± 0.9 | 104 ± 10 | 32.5 ± 1.3 | 66.0 ± 3.0 | 8.2 ± 0.3 | 30.0 ± 1.0 | 5.9 ± 0.2 |
| KS11_2020 | 12.2 ± 1.3 | 16.0 ± 2.0 | 1.7 ± 0.3 | 18.5 ± 1.1 | 14.4 ± 2.6 | 113 ± 22 | 33.2 ± 4.5 | 69.0 ± 3.0 | 9.0 ± 0.2 | 32.0 ± 1.0 | 6.5 ± 0.4 |
| HPAE3_2010 | 13.4 ± 0.4 | 17.0 ± 1.0 | 1.6 ± 0.2 | 27.2 ± 0.8 | 14.0 ± 0.7 | 170 ± 15 | 40.8 ± 1.3 | 85.0 ± 3.0 | 10.5 ± 0.4 | 38.0 ± 2.0 | 7.6 ± 0.4 |
| HPAE3_2011 | 13.7 ± 0.5 | 14.0 ± 1.0 | 1.2 ± 0.2 | 22.5 ± 1.4 | 11.2 ± 0.4 | 103 ± 11 | 36.1 ± 1.7 | 75.0 ± 3.0 | 9.3 ± 0.3 | 33.0 ± 1.0 | 6.6 ± 0.3 |
| HPAE3_2012 | 14.7 ± 2.8 | 17.0 ± 1.0 | 1.3 ± 0.3 | 23.7 ± 2.0 | 10.3 ± 1.1 | 113 ± 12 | 39.9 ± 4.1 | 78 ± 7 | 9.9 ± 0.9 | 36.0 ± 3.0 | 7.0 ± 0.6 |
| HPAE3_2013 | 13.9 ± 2.2 | 16.0 ± 2.0 | 1.2 ± 0.3 | 25.4 ± 1.8 | 10.1 ± 1.0 | 96 ± 12 | 45.5 ± 4.1 | 90 ± 7 | 11.4 ± 0.8 | 41.0 ± 3.0 | 8.1 ± 0.6 |
| HPAE3_2014 | 15.6 ± 1.7 | 18.0 ± 1.0 | 1.6 ± 0.2 | 23.5 ± 0.8 | 10.4 ± 0.9 | 130 ± 13 | 40.3 ± 2.0 | 79.0 ± 3.0 | 10 ± 0.4 | 37.0 ± 1.0 | 7.0 ± 0.3 |
| HPAE3_2015 | 15.8 ± 1.6 | 19.0 ± 1.0 | 1.4 ± 0.4 | 25.6 ± 2.7 | 10.8 ± 0.9 | 150 ± 15 | 43 ± 3.4 | 86 ± 6 | 10.5 ± 0.7 | 38.0 ± 2.0 | 7.6 ± 0.6 |
| HPAE3_2016 | 15.0 ± 1.0 | 19.0 ± 1.0 | 1.7 ± 0.3 | 28.0 ± 1.6 | 16.0 ± 1.0 | 126 ± 10 | 43.5 ± 2.9 | 88 ± 6 | 10.9 ± 0.7 | 40.0 ± 2.0 | 7.6 ± 0.5 |
| HPAE3_2017 | 15.6 ± 1.1 | 19.0 ± 1.0 | 1.8 ± 0.3 | 23.6 ± 0.9 | 14.4 ± 0.8 | 123 ± 12 | 35.3 ± 1.2 | 69.0 ± 3.0 | 8.5 ± 0.3 | 31.0 ± 1.0 | 6.0 ± 0.2 |
| KS8_2017 | 15.6 ± 1.0 | 19.0 ± 2.0 | 1.7 ± 0.3 | 21.8 ± 1.1 | 13.1 ± 0.9 | 102 ± 15 | 33.7 ± 1.6 | 67.0 ± 2.0 | 8.3 ± 0.3 | 30.0 ± 1.0 | 5.8 ± 0.3 |

Table A.6 Mass fractions (mg kg^-1^ or µg kg^-1^) of the size fraction <20 µm and the percentages of the <20 µm fraction of each sample. All given errors correspond to expanded uncertainties (U (k=2)).

|  | **Eu/mg kg^-1^** | **Gd/mg kg^-1^** | **Tb/mg kg^-1^** | **Dy/mg kg^-1^** | **Ho/mg kg^-1^** | **Er/mg kg^-1^** | **Tm/µg kg^-1^** | **Yb/mg kg^-1^** | **Lu/µg kg^-1^** | **Ta/µg kg^-1^** | **<20 µm/%** |
| --- | --- | --- | --- | --- | --- | --- | --- | --- | --- | --- | --- |
| URST1_2010 | 1.30 ± 0.08 | 5.9 ± 0.3 | 900 ± 40 | 5.3 ± 0.2 | 1010 ± 50 | 2.9 ± 0.1 | 400 ± 20 | 2.5 ± 0.2 | 350 ± 20 | 1000 ± 100 | 1.1 |
| URST1_2011 | 1.32 ± 0.06 | 6.0 ± 0.3 | 910 ± 40 | 5.3 ± 0.2 | 1000 ± 40 | 3.0 ± 0.1 | 400 ± 20 | 2.5 ± 0.2 | 350 ± 30 | 1100 ± 100 | 1.2 |
| URST1_2012 | 1.52 ± 0.06 | 6.2 ± 0.3 | 910 ± 30 | 5.4 ± 0.2 | 1030 ± 40 | 2.9 ± 0.1 | 370 ± 20 | 2.4 ± 0.2 | 380 ± 20 | 900 ± 100 | 7.5 |
| URST1_2013 | 1.61 ± 0.07 | 6.6 ± 0.2 | 970 ± 40 | 5.7 ± 0.1 | 1100 ± 40 | 3.1 ± 0.1 | 380 ± 20 | 2.5 ± 0.1 | 400 ± 20 | 900 ± 100 | 5.6 |
| URST1_2014 | 1.67 ± 0.13 | 6.9 ± 0.6 | 1010 ± 90 | 6.1 ± 0.5 | 1140 ± 80 | 3.2 ± 0.3 | 400 ± 30 | 2.6 ± 0.2 | 430 ± 50 | 900 ± 100 | 5.2 |
| URST1_2015 | 1.82 ± 0.26 | 7.6 ± 1.0 | 1110 ± 150 | 6.6 ± 0.8 | 1260 ± 170 | 3.5 ± 0.5 | 410 ± 40 | 2.7 ± 0.3 | 480 ± 70 | 1000 ± 200 | 12.2 |
| URST1_2016 | 1.12 ± 0.11 | 4.6 ± 0.3 | 720 ± 50 | 4.3 ± 0.3 | 830 ± 50 | 1.9 ± 0.1 | 270 ± 20 | 2.2 ± 0.2 | 320 ± 20 | 1100 ± 100 | 6.5 |
| URST1_2016 | 1.12 ± 0.11 | 4.6 ± 0.3 | 720 ± 50 | 4.3 ± 0.3 | 830 ± 50 | 1.9 ± 0.1 | 270 ± 20 | 2.2 ± 0.2 | 320 ± 20 | 1100 ± 100 | 6.7 |
| URST1_2017 | 1.69 ± 0.10 | 6.9 ± 0.3 | 1010 ± 20 | 6.0 ± 0.4 | 1130 ± 50 | 3.2 ± 0.1 | 350 ± 20 | 2.4 ± 0.2 | 430 ± 20 | 900 ± 100 | 7.0 |
| URST1_2019 | 1.38 ± 0.03 | 5.7 ± 0.1 | 840 ± 40 | 4.9 ± 0.2 | 940 ± 60 | 2.6 ± 0.1 | 360 ± 20 | 2.4 ± 0.2 | 340 ± 20 | 900 ± 100 | 3.9 |
| URST1_2020 | 1.25 ± 0.27 | 5.1 ± 1.2 | 780 ± 160 | 4.3 ± 0.2 | 830 ± 30 | 2.3 ± 0.4 | 310 ± 50 | 2.3 ± 0.3 | 350 ± 50 | 1000 ± 100 | 3.6 |
| Ti17_2010 | 1.38 ± 0.09 | 5.9 ± 0.3 | 880 ± 50 | 5.0 ± 0.2 | 940 ± 40 | 2.7 ± 0.1 | 370 ± 30 | 2.3 ± 0.2 | 320 ± 20 | 900 ± 100 | - |
| Ti17_2011 | 1.31 ± 0.07 | 5.7 ± 0.3 | 850 ± 50 | 5.0 ± 0.4 | 930 ± 80 | 2.7 ± 0.2 | 360 ± 30 | 2.2 ± 0.2 | 330 ± 30 | 900 ± 100 | - |
| Ti17_2013 | 1.23 ± 0.08 | 5.3 ± 0.4 | 800 ± 50 | 4.7 ± 0.3 | 880 ± 40 | 2.6 ± 0.2 | 360 ± 30 | 2.3 ± 0.2 | 320 ± 30 | 900 ± 100 | 2.7 |
| Ti17_2014 | 1.54 ± 0.23 | 6.7 ± 0.9 | 1020 ± 140 | 6.0 ± 0.9 | 1150 ± 160 | 3.4 ± 0.5 | 460 ± 70 | 2.9 ± 0.5 | 410 ± 60 | 1100 ± 100 | 2.0 |
| Ti17_2015 | 1.42 ± 0.15 | 6.3 ± 0.6 | 960 ± 90 | 5.7 ± 0.7 | 1090 ± 120 | 3.2 ± 0.3 | 430 ± 40 | 2.7 ± 0.3 | 390 ± 50 | 1000 ± 200 | 6.1 |
| Ti17_2016 | 1.28 ± 0.06 | 5.1 ± 0.3 | 770 ± 30 | 4.6 ± 0.2 | 880 ± 30 | 1.9 ± 0.1 | 260 ± 10 | 2.3 ± 0.2 | 330 ± 10 | 1200 ± 100 | 15.4 |
| Ti17_2016 | 1.28 ± 0.06 | 5.1 ± 0.3 | 770 ± 30 | 4.6 ± 0.2 | 880 ± 30 | 1.9 ± 0.1 | 260 ± 10 | 2.3 ± 0.2 | 330 ± 10 | 1200 ± 100 | 1.4 |
| Ti17_2016 | 1.28 ± 0.06 | 5.1 ± 0.3 | 770 ± 30 | 4.6 ± 0.2 | 880 ± 30 | 1.9 ± 0.1 | 260 ± 10 | 2.3 ± 0.2 | 330 ± 10 | 1200 ± 100 | 5.7 |
| Ti17_2017 | 1.28 ± 0.06 | 5.7 ± 0.3 | 840 ± 60 | 5.0 ± 0.3 | 950 ± 80 | 2.9 ± 0.2 | 380 ± 30 | 2.4 ± 0.2 | 340 ± 20 | 1000 ± 100 | 10.9 |
| Ti17_2018 | 1.84 ± 0.18 | 7.8 ± 0.6 | 1170 ± 90 | 6.9 ± 0.7 | 1310 ± 130 | 3.5 ± 0.1 | 480 ± 20 | 3.3 ± 0.4 | 500 ± 50 | 1100 ± 200 | 1.7 |
| Ti17_2019 | 1.36 ± 0.07 | 5.6 ± 0.3 | 820 ± 40 | 4.6 ± 0.2 | 890 ± 50 | 2.2 ± 0.1 | 310 ± 30 | 2.4 ± 0.1 | 350 ± 20 | 1100 ± 100 | 18.5 |
| Ti17_2020 | 1.18 ± 0.04 | 4.8 ± 0.2 | 690 ± 20 | 4.0 ± 0.2 | 760 ± 30 | 2.0 ± 0.1 | 280 ± 20 | 2.0 ± 0.1 | 280 ± 20 | 900 ± 100 | 3.9 |
| Ti7_2010 | 1.45 ± 0.08 | 6.8 ± 0.2 | 1040 ± 50 | 6.2 ± 0.5 | 1170 ± 70 | 3.5 ± 0.3 | 460 ± 50 | 2.9 ± 0.3 | 410 ± 20 | 1200 ± 200 | 1.0 |
| Ti7_2011 | 1.50 ± 0.08 | 6.9 ± 0.4 | 1080 ± 80 | 6.5 ± 0.3 | 1240 ± 70 | 3.6 ± 0.2 | 490 ± 30 | 3.0 ± 0.2 | 430 ± 20 | 1100 ± 100 | 0.9 |
| Ti7_2012 | 1.49 ± 0.08 | 6.7 ± 0.4 | 1060 ± 60 | 6.3 ± 0.4 | 1210 ± 70 | 3.5 ± 0.2 | 480 ± 40 | 2.9 ± 0.2 | 410 ± 10 | 900 ± 100 | 6.0 |
| Ti7_2017 | 1.82 ± 0.39 | 7.8 ± 1.8 | 1160 ± 230 | 6.7 ± 1.3 | 1280 ± 250 | 3.4 ± 0.6 | 470 ± 80 | 3.1 ± 0.6 | 460 ± 70 | 900 ± 100 | 4.7 |
| Ti7_2018 | 1.65 ± 0.07 | 6.8 ± 0.2 | 1010 ± 40 | 5.8 ± 0.2 | 1120 ± 40 | 3.0 ± 0.1 | 420 ± 20 | 2.9 ± 0.2 | 430 ± 20 | 1000 ± 100 | 5.6 |
| Ti7_2019 | 1.47 ± 0.08 | 6.3 ± 0.3 | 940 ± 40 | 5.6 ± 0.3 | 1060 ± 50 | 3.0 ± 0.1 | 410 ± 20 | 2.7 ± 0.1 | 390 ± 10 | 900 ± 100 | 3.5 |
| Ti19_2014 | 1.33 ± 0.09 | 5.8 ± 0.4 | 890 ± 50 | 5.3 ± 0.3 | 1000 ± 70 | 3.0 ± 0.2 | 410 ± 40 | 2.5 ± 0.1 | 360 ± 30 | 1100 ± 100 | 3.4 |
| Ti19_2015 | 1.08 ± 0.51 | 5.0 ± 1.5 | 730 ± 280 | 4.2 ± 2.3 | 790 ± 440 | 2.4 ± 1.3 | 310 ± 190 | 2.0 ± 1.2 | 300 ± 130 | 1000 ± 100 | 11.9 |
| KS11_2010 | 1.78 ± 0.07 | 8.6 ± 0.4 | 1180 ± 60 | 6.6 ± 0.2 | 1250 ± 50 | 3.6 ± 0.2 | 510 ± 30 | 3.4 ± 0.2 | 540 ± 30 | 1400 ± 100 | 22.4 |
| KS11_2011 | 1.57 ± 0.04 | 7.1 ± 0.3 | 990 ± 40 | 5.7 ± 0.2 | 1080 ± 30 | 3.1 ± 0.2 | 400 ± 30 | 2.7 ± 0.2 | 440 ± 30 | 1200 ± 100 | 23.3 |
| KS11_2012 | 1.30 ± 0.08 | 6.0 ± 0.3 | 870 ± 60 | 4.9 ± 0.2 | 910 ± 40 | 2.6 ± 0.2 | 360 ± 20 | 2.3 ± 0.1 | 330 ± 10 | 1000 ± 100 | 53.7 |
| KS11_2013 | 1.47 ± 0.11 | 5.8 ± 0.2 | 850 ± 90 | 4.9 ± 0.5 | 950 ± 90 | 2.7 ± 0.3 | 340 ± 20 | 2.2 ± 0.2 | 370 ± 40 | 1000 ± 300 | 63.1 |
| KS11_2014 | 1.32 ± 0.16 | 5.3 ± 0.3 | 800 ± 70 | 5.0 ± 0.8 | 930 ± 140 | 2.8 ± 0.5 | 390 ± 90 | 2.4 ± 0.6 | 320 ± 40 | 700 ± 400 | 83.9 |
| KS11_2015 | 1.37 ± 0.05 | 5.5 ± 0.3 | 800 ± 30 | 4.6 ± 0.2 | 890 ± 40 | 2.5 ± 0.1 | 320 ± 20 | 2.1 ± 0.2 | 360 ± 20 | 1000 ± 100 | 47.3 |
| KS11_2016 | 1.12 ± 0.16 | 4.5 ± 0.6 | 690 ± 80 | 4.1 ± 0.4 | 790 ± 60 | 2.2 ± 0.1 | 310 ± 30 | 2.1 ± 0.2 | 330 ± 20 | 1100 ± 100 | 35.1 |
| KS11_2016 | 1.12 ± 0.16 | 4.5 ± 0.6 | 690 ± 80 | 4.1 ± 0.4 | 790 ± 60 | 2.2 ± 0.1 | 310 ± 30 | 2.1 ± 0.2 | 330 ± 20 | 1100 ± 100 | 43.2 |
| KS11_2017 | 1.20 ± 0.04 | 5.2 ± 0.2 | 750 ± 30 | 4.3 ± 0.2 | 820 ± 40 | 2.4 ± 0.1 | 330 ± 20 | 2.1 ± 0.2 | 300 ± 10 | 900 ± 100 | 74.4 |
| KS11_2020 | 1.30 ± 0.08 | 5.4 ± 0.3 | 780 ± 80 | 4.5 ± 0.4 | 860 ± 80 | 2.2 ± 0.2 | 310 ± 30 | 2.3 ± 0.3 | 350 ± 40 | 1100 ± 200 | 51.6 |
| HPAE3_2010 | 1.45 ± 0.07 | 6.7 ± 0.3 | 980 ± 60 | 5.6 ± 0.3 | 1060 ± 50 | 3.1 ± 0.1 | 420 ± 20 | 2.6 ± 0.2 | 380 ± 30 | 1100 ± 100 | 2.5 |
| HPAE3_2011 | 1.26 ± 0.08 | 5.8 ± 0.2 | 830 ± 40 | 4.8 ± 0.3 | 910 ± 40 | 2.6 ± 0.1 | 360 ± 10 | 2.3 ± 0.2 | 330 ± 20 | 900 ± 100 | 8.7 |
| HPAE3_2012 | 1.55 ± 0.14 | 6.2 ± 0.6 | 900 ± 100 | 5.2 ± 0.5 | 980 ± 80 | 2.8 ± 0.2 | 290 ± 20 | 1.9 ± 0.2 | 380 ± 40 | 900 ± 200 | 18.2 |
| HPAE3_2013 | 1.64 ± 0.17 | 7.0 ± 0.5 | 1000 ± 100 | 5.8 ± 0.6 | 1110 ± 120 | 3.2 ± 0.4 | 300 ± 30 | 2.1 ± 0.3 | 460 ± 60 | 900 ± 100 | 27.0 |
| HPAE3_2014 | 1.63 ± 0.05 | 6.3 ± 0.4 | 930 ± 50 | 5.4 ± 0.3 | 1050 ± 70 | 3.0 ± 0.2 | 280 ± 30 | 1.8 ± 0.1 | 420 ± 40 | 900 ± 100 | 37.6 |
| HPAE3_2015 | 1.66 ± 0.12 | 6.7 ± 0.5 | 980 ± 60 | 5.9 ± 0.4 | 1120 ± 90 | 3.2 ± 0.2 | 270 ± 30 | 1.9 ± 0.1 | 460 ± 40 | 1000 ± 100 | 31.9 |
| HPAE3_2016 | 1.47 ± 0.04 | 6.8 ± 0.4 | 960 ± 40 | 5.5 ± 0.2 | 1050 ± 50 | 2.9 ± 0.2 | 470 ± 20 | 3.2 ± 0.2 | 410 ± 20 | 1100 ± 100 | 19.0 |
| HPAE3_2017 | 1.30 ± 0.07 | 5.4 ± 0.1 | 790 ± 30 | 4.6 ± 0.1 | 890 ± 40 | 2.5 ± 0.1 | 380 ± 20 | 2.5 ± 0.1 | 340 ± 20 | 1000 ± 100 | 26.5 |
| KS8_2017 | 1.29 ± 0.07 | 5.2 ± 0.2 | 750 ± 40 | 4.4 ± 0.3 | 840 ± 40 | 2.4 ± 0.1 | 340 ± 20 | 2.2 ± 0.3 | 330 ± 30 | 1000 ± 100 | 69.0 |

Figure A.1 REE-PAAS pattern of two representative sample station of the two regions at the beginning (2010) and the end (2020) of the observed time series (North red, South blue).
